# Neurointensive care of traumatic brain injury in the elderly—age-specific secondary insult levels and optimal physiological levels to target need to be defined

**DOI:** 10.1007/s00701-021-05047-z

**Published:** 2021-11-10

**Authors:** Samuel Lenell, Anders Lewén, Timothy Howells, Per Enblad

**Affiliations:** Department of Neuroscience/Neurosurgery, Section of Neurosurgery, Uppsala University, Uppsala University Hospital, 751 85 Uppsala, Sweden

**Keywords:** Traumatic brain injury, Elderly, Outcome, Secondary insults, Geriatric neurointensive care, Neurointensive care monitoring

## Abstract

**Background:**

Elderly patients with traumatic brain injury increase. Current targets and secondary insult definitions during neurointensive care (NIC) are mostly based on younger patients. The aim was therefore to study the occurrence of predefined secondary insults and the impact on outcome in different ages with particular focus on elderly.

**Methods:**

Patients admitted to Uppsala 2008–2014 were included. Patient characteristics, NIC management, monitoring data, and outcome were analyzed. The percentage of monitoring time for *ICP*, *CPP*, *MAP*, and *SBP* above-/below-predefined thresholds was calculated.

**Results:**

Five hundred seventy patients were included, 151 elderly ≥ 65 years and 419 younger 16–64 years. Age ≥ 65 had significantly higher percentage of *CPP* > 100, *MAP* > 120, and *SBP* > 180 and age 16–64 had higher percentage of *ICP* ≥ 20, *CPP* ≤ 60, and *MAP* ≤ 80. Age ≥ 65 contributed independently to the different secondary insult patterens. When patients in all ages were analyzed, low percentage of CPP > 100 and SBP > 180, respectively, was significant predictors of favorable outcome and high percentage of *ICP* ≥ 20, *CPP* > 100, *SBP* ≤ 100, and *SBP* > 180, respectively, was predictors of death. Analysis of age interaction showed that patients ≥ 65 differed and had a higher odds for favorable outcome with large proportion of good monitoring time with *SBP* > 180.

**Conclusions:**

Elderly ≥ 65 have different patterns of secondary insults/physiological variables, which is independently associated to age. The finding that SBP > 180 increased the odds of favorable outcome in the elderly but decreased the odds in younger patients may indicate that blood pressure should be treated differently depending on age.

**Supplementary Information:**

The online version contains supplementary material available at 10.1007/s00701-021-05047-z.

## Introduction

The introduction of neurointensive care (NIC), with focused efforts of avoiding secondary insults, has contributed to an increase of favorable outcome for traumatic brain injury (TBI) patients [2, 3, 8, 23, 27]. Despite this improvement, TBI still constitutes a large health problem. The magnitude of the problem is illustrated by a recent overview of TBI in Europe showing that the incidence of hospitalized TBI patients was 278.2/100 000 in 2012 (Sweden 2013, 451.5/100 000) and the mortality rate was 11.7/100 000 (Sweden 2013, 9.0/100 000) [21]. Despite that elderly (age ≥ 65 years) constituted only 29% of the hospitalized TBI patients, they contributed to 55% of the mortality [21]. It is obvious that the management of elderly TBI patients will be a tremendous challenge for the future for many reasons. In addition to higher mortality rate in the elderly [10, 17, 21], the elderly are an increasing part of the population and they live more active lives than before [10, 17, 18]. Traditionally, there has been some reluctance to treat these patients due to the previous experience of bad outcome, but more recently, larger numbers of elderly are treated [25, 30, 32, 33, 38]. Hence, it is urgent to obtain more knowledge about the optimal treatment of elderly TBI patients.

The NIC of patients with TBI in general is mostly based on data from younger patients and there is insufficient research in the elderly despite the change in population structure [9]. For example, large clinical TBI trials have often been made with age > 65 years as an exclusion criteria [5, 14, 19, 24, 26]. Although the secondary insult prevention concept is one of the main reasons for the improvement of NIC, it is likely that both critical and optimal threshold levels differ between ages. This is underlined by studies in elderly patients with severe subarachnoid hemorrhage showing that the occurrence of defined secondary insults and the impact on outcome was age-dependent [31]. In order to optimize the NIC of elderly TBI patients, it is desirable to identify the critical threshold levels for secondary insults and the optimal threshold levels to target, specifically in the older ages.

The aim of this investigation was therefore to study the occurrence of predefined secondary insults and the impact of outcome in different ages with particular focus on the elderly.

## Material and methods

### Patient selection and data collection

All TBI patients ≥ 16 years old receiving NIC at Uppsala University Hospital between 2008 and 2014 were retrieved from the Uppsala TBI registry [28]. In total, 663 patients were identified. The following patients were excluded as follows: recovery within 24 h after admission (11 patients), admission more than 5 days after trauma (23 patients), bilateral wide and unresponsive pupils (15 patients) or Glasgow coma scale score 3 and one wide pupil on admission (1 patient) (patients with probable predestined fatal/unfavorable clinical course judged in general not possible to treat [1, 4]), gunshot to head (4) and lost to follow up (39 patients). Finally, 570 patients remained to be analyzed.

### Demographics and NIC management data

Demographic data and information about NIC management were obtained from the Uppsala TBI registry [28]. The following parameters were studied as follows: age, sex, primary or secondary transfer, Glasgow coma scale motor score (GCS M) on admission, type of injury, presence of multiple injuries, trauma under the influence of alcohol or drugs, cause of trauma, medical history (brain injury/disease, previous traumatic brain injury, diabetes mellitus, hypertension/cardiovascular disease (CVD), use of anticoagulants/antiplatelets), craniotomy, decompressive craniectomy, intracranial pressure monitoring, and mechanical ventilation. The type of injury was assessed on the initial CT-scan (dominating type of injury and Marshall CT score [22]).

### Physiological data

Trended minute-by-minute data (median values of 5 samples during each sampled minute) was collected in real time from the Philips monitors in our ICU using the Odin software [12]. The Philips monitors forward the data to a central database within the hospital, which is queried by the Odin server to extract the relevant data which is stored centrally and displayed on Odin client systems at the ICU bedspaces. The patient data stored and processed by the Odin software is also kept within the hospital firewall. The trended data used in this study were preprocessed with median filters to detect sudden spikes that appeared to be non-physiological, and a specialized algorithm detected sudden drops to a constant value (usually zero). The data were further subject to manual review to verify, and if necessary correct, the automatic procedures. Time gaps from, e.g., radiology examination and surgical procedure were automatically excluded by the Odin software. The monitoring time left was defined as good monitoring time (GMT).

For the purpose of evaluating physiological NIC monitoring data (intra cranial pressure, ICP; cerebral perfusions pressure, *CPP*; mean arterial pressure, *MAP*; and systolic blood pressure, SBP), *GMT* data from the start of monitoring to the end of the seventh monitoring day was studied.For ICP and *CPP* analyses, at least 12 h of *ICP* data was required. Using the Odin software, the proportions of good monitoring time (%*GMT*) spent above-/below-predefined threshold levels were calculated for *ICP* ≥ 20, *CPP* ≤ 60, *CPP* > 100, *MAP* ≤ 80, *MAP* > 120, *SBP* ≤ 100, and *SBP* > 180. The thresholds originated mainly from our protocol treatment goals [8].

### Neurointensive care protocol

All patients were treated according to the same standardized treatment protocol [8]. Unconscious patients (GCS M ≤ 5) had mechanical ventilation. Patients on mechanical ventilation were kept sedated with propofol (Propofol-LipuroB; Braun Medical, Danderyd, Sweden) and received morphine for analgesia. They were initially moderately hyperventilated (PaCO_2_ 4.0–4.5 kPa) with the aim of normoventilation as soon as ICP allowed (*ICP* < 20 mmHg). Wake-up tests were performed regularly (usually 3–6 times/day unless severe ICP elevations) to assess neurological function. All unconscious patients (GCS *M* ≤ 5), regardless of age, had also ICP monitoring, except in the case of coagulopathy. An external ventricular drainage system (EVD) (with the pressure dome at the level of the lateral ventricles) was the first choice and an intraparenchymal pressure device was chosen if the ventricles were compressed. Arterial blood pressure was measured with the pressure dome at heart level. Prophylactic anticonvulsants was not used. The treatment goals according to the standardized management protocol were as follows: *ICP* < 20 mmHg, *SBP* > 100 mmHg, central venous pressure (CVP) 0–5 mmH_2_O, *CPP* > 60 mmHg, blood glucose 5–10 mmol/L, normovolemia, Pa0_2_ > 12 kPa, electrolytes within normal ranges, and body temperature < 38 °C.

Mass lesions in unconscious patients were evacuated.

Raised ICP was treated in a stepwise fashion. If ICP increased ≥ 20 mmHg without mass lesions, cerebrospinal fluid (CSF) was drained from the EVD. Initially small volumes (1–2 ml) were drained intermittently, when there were risk of expanding hematomas and brain swelling. Later CSF was drained using an open system against a pressure level of 15–20 mmHg if needed. If raised ICP persisted, the treatment was escalated with no wake-up tests, continuous sedation with propofol, and stress reduction with ß1-antagonist metoprolol (Seloken®, AstraZeneca AB Södertälje, Sweden) (0.2–0.3 mg/kg/24 h as an infusion) and α2-agonist clonidin (Catapresan®, BoehingerIngelheim AB Stockholm Sweden) (0.5–1.0 μg/kg × 8 or the same dose as an infusion).Thiopental coma treatment and/or decompressive craniectomy were last tier treatment option but were initiated more restrictively in the elderly.

### Outcome

The NIC mortality was assessed. Follow-up was done after 6 months, using the extended Glasgow outcome scale (GOSE), by structured telephone interviews done by a few selected persons[34, 39].

### Statistics

Differences in the characteristics between age groups were analyzed with Pearsons Chi 2 test.

Mann-Withney *U* test was used to compare occurrence of secondary insults between the age groups.

Multiple linear regression analysis was done to examine if age ≥ 65 years and admission variables as gender, GCS M, other injuries, extracerebral hematoma, and contusions contributed to the %*GMT* above/below secondary insult thresholds for the physiological variables.

To evaluate if the %GMT above/below secondary insult thresholds for the physiological variables was associated with outcome, univariate logistic regression analysises were made with favorable outcome (GOSE 5–8) and survival (GOSE 2–8) as dependent variables. To evaluate whether associations differed by age (age 16–64 vs age ≥ 65), multiple logistic regression models were fitted including age, a physiological variable and age by physiological variable interaction as independent variables. The odds ratios (ORs) for physiological variables are reported for each age-group, regardless of the significance of interaction.

*p* < 0.05 was considered statistically significant. All statistical analyses were carried out in IBM SPSS Statistics for Windows except for Pearsons Chi 2 which was done with Microsoft Excel 365.

## Results

### Admission characteristics

For all patients, the mean age was 49.7 years (range 16–94). The age distribution showed one peak at around 20 years of age and another peak around 60–65 years of age (Supplementary Information 1). There were 151 patients ≥ 65 years (mean 72.3 range 65–87) and 419 between 16 and 64 (mean 41.5 range 16–64) years of age. Patient characteristics from admission are presented in Table 1. When the age groups of ≥ 65 years and 16–64 years were compared, the older patients showed significantly larger proportions of women ( 28.5% vs 19.6%), fall accidents (80.1 vs 42.0%), previous brain injury/disease (22.5% vs 11.0%), diabetes mellitus (18.5 vs 6.2%), hypertension/cerebrovascular disease (58.3% vs 13.8%), ongoing treatment with anticoagulants/antiplatelets (43.0% vs 7.9%), and significantly smaller proportions of patients admitted from other hospitals (67.5% vs 82.3%), multiple injuries (17.9% vs 47.0%), influence of drugs/alcohol (14.6% vs 34.1%), vehicle accidents (7.3% vs 33.2%), and sports injury (0.7% vs 4.3%). Regarding the dominating type of injury assessed on initial CT, the older patients had significantly larger proportion of acute subdural hematoma (51.7% vs 20.5%) and smaller proportion of diffuse axonal injury (DAI) (0.0% vs 8.6%) and epidural hematoma (0.7% vs 11.5%) (Table 2). There was no difference between the age groups in GCS M on admission (Table 1 and Supplementary Information 2).

**Table 1:** Patient characteristics by age group

	All		Age 16–64		Age ≥ 65		Age 16–64 vs ≥ 65^a^
	*n*	%	*n*	%	*n*	%	*p*
No. of patients	570		419		151		
Referrals from other hospitals	447	78.4	345	82.3	102	67.5	< 0.001 ***
Sex (female)	125	21.9	82	19.6	43	28.5	0.023 *
GCS M ≥ 4 on admission	518	90.9	382	91.2	136	90.1	0.687
GCS M ≤ 5 on admission	310	54.4	233	55.6	77	51.0	0.329
Multiple injuries	224	39.3	197	47.0	27	17.9	< 0.001 ***
Under the influence of drugs/alcohol at trauma (confirmed)	165	28.9	143	34.1	22	14.6	< 0.001 ***
Cause of trauma							
Bicycle accident	16	2.8	14	3.3	2	1.3	
Fall accident	297	52.1	176	42.0	121	80.1	< 0.001 ***
Vehicle accident	150	26.3	139	33.2	11	7.3	< 0.001 ***
Pedestrian hit by vehicle	24	4.2	17	4.1	7	4.6	0.762
Assault	33	5.8	30	7.2	3	2.0	0.020
Sports injury	19	3.3	18	4.3	1	0.7	0.033 *
Other	31	5.4	25	6.0	6	4.0	0.355
Medical history							
Brain injury/disease previously	80	14.0	46	11.0	34	22.5	< 0.001 ***
Traumatic brain injury previously	18	3.2	11	2.6	7	4.6	
Diabetes mellitus	54	9.5	26	6.2	28	18.5	< 0.001 ***
Hypertension/CVD	146	25.6	58	13.8	88	58.3	< 0.001 ***
Anticoagulants/Antiplatelets	98	17.2	33	7.9	65	43.0	< 0.001 ***
Ethylism	126	22.1	95	22.7	31	20.5	0.586

^a^Pearsons Chi 2 test, *p < 0.05, **p < 0.01, and ***p < 0.001

**Table 2:** Radiological characteristics by age group

	All		Age 16–64		Age ≥ 65		Age 16–64 vs ≥ 65
	*n*	%	*N*	%	*n*	%	*p*
No. of patients	570		419		151		
Dominating CT finding							
ASDH	164	28.8	86	20.5	78	51.7	< 0.001 ***
EDH	49	8.6	48	11.5	1	0.7	< 0.001 ***
Contusions	171	30.0	132	31.5	39	25.8	0.192
DAI	36	6.3	36	8.6	0	0.0	< 0.001 ***
Mixed	68	11.9	53	12.6	15	9.9	0.378
Impression fracture	12	2.1	11	2.6	1	0.7	
Traumatic SAH	53	9.3	38	9.1	15	9.9	0.754
Normal	6	1.1	6	1.4	0	0.0	
Other	11	1.9	9	2.1	2	1.3	
Initial CT Marshall classification							
Diffuse injury	393	68.9	325	77.6	68	45.0	< 0.001 ***
Diffuse injury I	6	1.1	5	1.2	1	0.7	
Diffuse injury II	279	48.9	236	56.3	43	28.5	< 0.001 ***
Diffuse injury III	82	14.4	69	16.5	13	8.6	0.018 *
Diffuse injury IV	26	4.6	15	3.6	11	7.3	0.061
Focal mass lesion	117	20.5	94	22.4	23	15.2	0.060
Evacuated mass lesion	126	22.1	69	16.5	57	37.7	< 0.001 ***
Nonevacuated mass lesion	51	8.9	25	6.0	26	17.2	< 0.001 ***

^a^Pearsons Chi 2 test, *p* < 0.05, **p < 0.01, and ***p < 0.001

### NIC management and surgery

There were no significant differences between the age groups ≥ 65 years and 16–64 years regarding ICP monitoring (55.0% vs 62.5%) and mechanical ventilation (82.1% vs 77.3%) (Table 3). The proportion of patients treated with thiopental were significantly smaller in the old age group (0.7% vs 7.9%) (Table 3). The old group had significantly more craniotomies compared to the younger group (47.7% vs 32.7%) (Table 3).

**Table 3:** Treatment characteristics by age group

	All		Age 16–64		Age ≥ 65		Age 16–64 vs ≥ 65^a^
	*N*	%	*n*	%	n	%	*p*
No. of patients	570		419		151		
Surgery							
Craniotomy at referring hospital	50	8.8	36	8.6	14	9.3	0.800
Craniotomy (yes/no)	209	36.7	137	32.7	72	47.7	0.001 **
Reasons for craniotomy^b^							
Extra cerebral hematoma	167	29.3	99	23.6	68	45.0	< 0.001 ***
EDH	35	6.1	34	8.1	1	0.7	0.001 **
ASDH	120	21.1	55	13.1	65	43.0	< 0.001 ***
Both (EDH + ASDH)	12	2.1	10	2.4	2	1.3	
Contusions	66	11.6	52	12.4	14	9.3	0.301
Hemicraniectomy	39	6.8	34	8.1	5	3.3	0.045 *
Multiple surgeries (yes/no)	61	10.7	43	10.3	18	11.9	0.572
Management, NIC							
ICP monitoring	345	60.5	262	62.5	83	55.0	0.103
EVD	65	11.4	47	11.2	18	11.9	0.816
Intraparenchymal pressure monitor	206	36.1	153	36.5	53	35.1	0.756
Both	74	13.0	62	14.8	12	7.9	0.032 *
Mean days with ICP monitoring	11.2		11.8		9.2		
Mechanical ventilation	448	78.6	324	77.3	124	82.1	0.218
Mean days ventilation	9.0		9.6		7.4		
Thiopenthal	34	6.0	33	7.9	1	0.7	0.001 **
Mean days with Thiopenthal	6.2		6.2		6		

^a^Pearsons Chi 2 test, *p < 0.05, **p < 0.01, and ***p < 0.001

^b^Multiple operations in some patients

### Physiological data

**Fig. 1:**
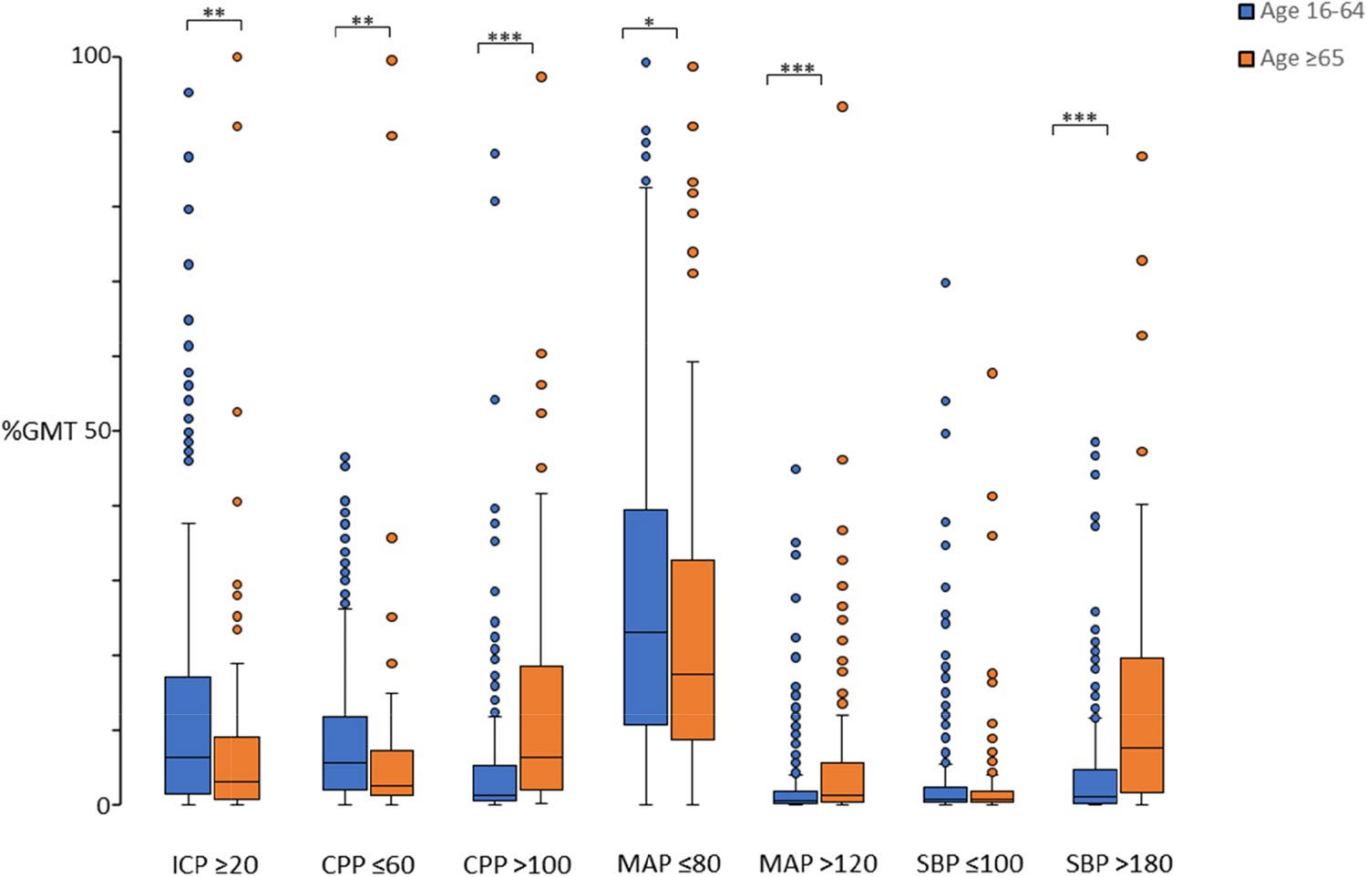
Proportion of good monitoring time (%*GMT*) for different insult variables by age group. In the box plots, the horizontal black line marks the median, boxes extend from the 25th to the 75th percentile, vertical extending lines denote adjacent values (i.e., the most extreme values within 1.5 interquartile range of the 25th and 75th percentile of each group) and the dots denote observations outside the range of adjacent values (outliers).Mann–Whitney *U* test, *p < 0.05, **p < 0.01, and ***p < 0.001

Monitoring information regarding number of patients for each physiological parameter and age group is presented in Table 4. When the occurrences of physiological variables were analyzed as median %*GMT* (Table 5 and Fig. 1), there were statistically significant differences between the age groups: age ≥ 65 years had significantly higher %*GMT* with *CPP* > 100, *MAP* > 120, and *SBP* > 180 and age 16–64 years had significantly higher %*GMT* with *ICP* ≥ 20, *CPP* ≤ 60, and *MAP* ≤ 80.

**Table 4:** Monitoring by age group

	All	Age 16–64		Age ≥ 65		16–64 vs ≥ 65^a^
	*N*	*n*	%	*n*	%	*p*
No. of patients	570	419		151		
*ICP*	333	253	60.38	80	52.98	0.114
*CPP*	332	252^e^	60.14	80	52.98	0.126
*MAP*	521	377	89.98	144	95.36	0.043 *
*SBP*	521	377	89.98	144	95.36	0.043 *

^a^Pearsons Chi 2 test, *p < 0.05

^e^Continuous MAP data was missing in one patient with ICP monitoring

**Table 5:** Occurrence of secondary insults by age group

Physiological parameter	All patients		Age 16–64		Age ≥ 65		16–64 vs ≥ 65^d^
	Median %GMT^c^	IQR %GMT^c^	Median %GMT^c^	IQR %GMT^c^	Median %GMT^c^	IQR %GMT^c^	*p*
*ICP* ≥ 20	5.26	1.28**–**15.46	6.26	1.39**–**17.01	3.14	0.73**–**9.05	0.005 **
*CPP* ≤ 60	4.72	1.60**–**11.02	5.52	2.05**–**11.79	2.51	1.16**–**1.94	0.001 **
*CPP* > 100	1.97	0.62**–**8.10	1.27	0.51**–**5.25	6.37	1.96**–**18.57	0.000 ***
*MAP* ≤ 80	21.92	9.63**–**38.20	23.01	10.67**–**39.49	17.51	8.75**–**32.68	0.040 *
*MAP* > 120	0.59	0.21**–**2.52	0.48	0.17**–**1.77	1.31	0.36**–**5.52	0.000 ***
*SBP* ≤ 100	0.75	0.25**–**2.20	0.75	0.25**–**2.39	0.71	0.25**–**1.83	0.499
*SBP* > 180	2.10	0.23**–**7.81	1.04	0.18**–**4.72	7.53	1.54**–**19.63	0.000 ***

^c^%*GMT* denotes percentage of good monitoring time above/below the thresholds

^d^Mann-Whitney *U* test, *p < 0.05, **p < 0.01, and ***p < 0.001

The multiple linear regression model with physiological variables as dependent variables and age ≥ 65 years, gender, GCS M, other injuries, extracerebral hematoma, and contusions as explanatory variables showed that age ≥ 65 years was an independent predictor for lower %GMT with *ICP* ≥ 20 and higher %*GMT* with *CPP* > 100, *MAP* > 120, and *SBP* > 180 (Table 6). Higher GCS M score was an independent predictor for low %GMT with *ICP* ≥ 20 and *CPP* ≤ 60 (Table 6). Other injuries were found to be an independent predictor for lower %*GMT* with *ICP* ≥ 20, *CPP* > 100, *MAP* > 120, and *SBP* > 180 and for higher %*GMT* with *MAP* ≤ 80 (Table 6). Females showed significanly lower %*GMT* with *SBP* > 180 and higher %*GMT* with *SBP* ≤ 100. (Table 6).

**Table 6:** Linear regression analysis of contribution from admission characteristics and age ≥ 65 to physiological variables

Physiological variable (%*GMT*)	Explanatory variable	level	*B*	*(95% CI)*	*p* value
*ICP* ≥ 20	Age ≥ 65	Yes	− 0.05	(− 0.10 to − 0.10)	0.016 *
	Sex (female)	Yes	− 0.04	(− 0.08 to 0.01)	0.130
	GCS Motor Score	Per score increase	− 0.02	(− 0.04 to − 0.01)	0.005 **
	Other injuries	Yes	− 0.07	(− 0.04 to − 0.01)	0.001 **
	Extracerebral hematoma	Yes	− 0.03	(− 0.08 to 0.01)	0.166
	Contusions	Yes	− 0.01	(− 0.05 to 0.04)	0.744
*CPP* ≤ 60	Age ≥ 65	Yes	− 0.02	(− 0.05 to 0.01)	0.176
	Sex (female)	Yes	0.01	(− 0.02 to 0.04)	0.594
	GCS Motor Score	Per score increase	− 0.01	(− 0.02 to 0.00)	0.046 *
	Other injuries	Yes	0.00	(− 0.03 to 0.02)	0.836
	Extracerebral hematoma	Yes	− 0.01	(− 0.04 to 0.03)	0.687
	Contusions	Yes	− 0.01	(− 0.04 to 0.02)	0.486
*CPP* > 100	Age ≥ 65	Yes	0.06	(0.03 to 0.09)	0.000 ***
	Sex (female)	Yes	0.00	(− 0.04 to 0.03)	0.846
	GCS Motor Score	Per score increase	0.00	(− 0.01 to 0.02)	0.610
	Other injuries	Yes	− 0.05	(− 0.08 to − 0.02)	0.000 ***
	Extracerebral hematoma	Yes	0.01	(− 0.02 to 0.05)	0.469
	Contusions	Yes	− 0.03	(− 0.07 to − 0.00)	0.043
*MAP* ≤ 80	Age ≥ 65	Yes	− 0.02	(− 0.06 to 0.02)	0.347
	Sex (female)	Yes	0.06	(0.01 to 0.10)	0.011 *
	GCS Motor Score	Per score increase	− 0.01	(− 0.02 to 0.01)	0.363
	Other injuries	Yes	0.09	(0.05 to 0.13)	0.000 ***
	Extracerebral hematoma	Yes	0.01	(− 0.04 to 0.05)	0.809
	Contusions	Yes	0.00	(− 0.05 to 0.05)	0.939
*MAP* > 120	Age ≥ 65	Yes	0.02	(0.01 to 0.04)	0.009 **
	Sex (female)	Yes	0.00	(− 0.02 to 0.01)	0.828
	GCS Motor Score	Per score increase	0.00	(0.00 to 0.008)	0.522
	Other injuries	Yes	− 0.03	(− 0.04 to − 0.02)	0.000 ***
	Extracerebral hematoma	Yes	0.00	(− 0.02 to 0.02)	0.964
	Contusions	Yes	0.00	(− 0.02 to 0.01)	0.636
*SBP* ≤ 100	Age ≥ 65	Yes	− 0.01	(− 0.02 to 0.01)	0.364
	Sex (female)	Yes	0.02	(0.01 to 0.04)	0.001 **
	GCS Motor Score	Per score increase	0.00	(− 0.01 to 0.00)	0.116
	Other injuries	Yes	− 0.01	(− 0.02 to 0.00)	0.119
	Extracerebral hematoma	Yes	0.00	(− 0.02 to 0.01)	0.732
	Contusions	Yes	− 0.01	(− 0.02 to 0.01)	0.455
*SBP* > 180	Age ≥ 65	Yes	0.08	(0.06 to 0.12)	0.000 ***
	Sex (female)	Yes	− 0.03	(− 0.05 to − 0.01)	0.001 **
	GCS Motor Score	Per score increase	0.00	(− 0.01 to 0.01)	0.474
	Other injuries	Yes	− 0.03	(− 0.05 to − 0.01)	0.002 **
	Extracerebral hematoma	Yes	0.00	(− 0.03 to 0.02)	0.745
	Contusions	Yes	0.01	(− 0.02 to 0.03)	0.657

Multivariate linear regression analyses of each physiological variables as dependent and age ≥ 65, sex, GCS motor score, other injuries, extracerebral hematoma, and contusions as explanatory variables. *p < 0.05, **p < 0.01, and ***p < 0.001. Positive *B* coefficients indicate that the increasing value of the explanatory variable are associated with a larger %GMT of the dependent variable. Negative *B* coefficients indicate that the increasing value of the explanatory variable are associated with a lower %GMT of the dependent variable

### Outcome

**Fig. 2:**
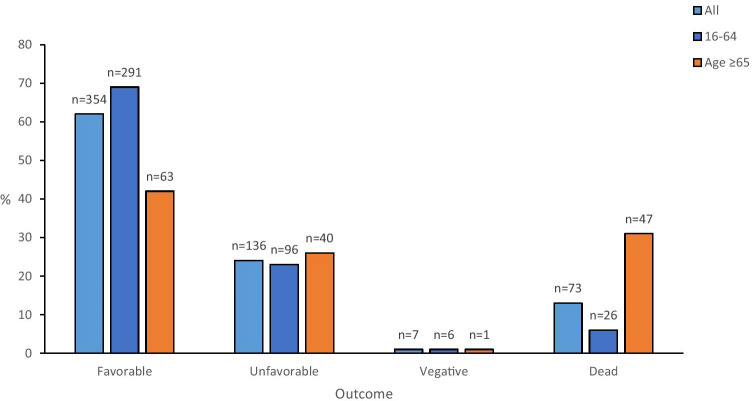
Clinical outcome at follow-up. Favorable outcome (GOSE 5–8), unfavorable (GOSE 3–4), vegetative (GOSE 2), and dead (GOSE 1). Each bar represents the percentage of outcome within its age group. Absolute number of patients in each bar is presented above

NIC mortality was higher in the old age group (≥ 65 years 8.6% and 16–64 years 2.4%, *p* < 0.001). Follow-up was made at 7 months in median (range 1–28, including patients who died before follow-up). For all ages, favorable outcome (GOSE 5–8) was observed in 62% (69% in 16–64 years and 42% in elderly) and 13% had died (6% in 16–64 years and 31% in elderly) (Fig. 2).

The results from the logistic regression analyses with favorable outcome and survival as dependent variables and physiological parameters as explanatory variables are presented in Table 7. Low %*GMT* with *CPP* > 100 and *SBP* > 180 were associated with a higher odds of favorable outcome. However, there was a statistically significant interaction between age and %*GMT* with *SBP* > 180 (*p* interaction = 0.025). The *OR* (per unit increase in %*GMT* with *SBP* > 180) was 2.07 (0.22–1731.66) in patients ≥ 65 years and − 0.03(0.00–0.57) in patients 16–64 years (Table 7). High %*GMT* with *ICP* ≥ 20, *CPP* > 100, *SBP* ≤ 100 were associated with a lower odds of survival (Table 7).

**Table 7:** Univariate logistic regression analysis of outcome in relation to physiological variables and the interaction^d^ with age

Physiological variable		All ages				Age 16–64				Age ≥ 65		Interaction	
(%***GMT***)	***OR***	(95% CI)	***p***	***OR***	(95% CI)		***p***	***OR***	(95% CI)		***p***	***p*** value^d^	
Model with favorable outcome as dependent													
* ICP* ≥ 20	0.33	(0.09**–**1.20)	0.093	0.16	(0.03**–**0.72)		0.017 *	1.34	(0.10**–**18.70)		0.827	0.167	
* CPP* ≤ 60	1.09	(0.17**–**7.01)	0.926	1.22	(0.10**–**14.49)		0.874	0.50	(0.02**–**12.37)		0.669	0.663	
* CPP* > 100	0.02	(0.00**–**0.19)	0.001 **	0.04	(0.00**–**0.74)		0.030 *	0.03	(0.00**–**1.20)		0.062	0.851	
* MAP* ≤ 80	2.04	(0.88**–**4.76)	0.097	2.36	(0.83**–**6.72)		0.107	0.84	(0.17**–**4.24)		0.833	0.294	
* MAP* > 120	0.17	(0.02**–**1.75)	0.136	0.42	(0.01–14.55)		0.628	0.47	(0.02–11.68)		0.645	0.959	
* SBP* ≤ 100	0.45	(0.04–5.56)	0.531	0.80	(0.04–17.88)		0.885	0.03	(0.00–21.97)		0.294	0.374	
* SBP* > 180	0.09	(0.02–0.53)	0.007 ***	0.03	(0.00–0.57)		0.020 *	2.07	(0.22–1731.66)		0.521	0.025 *	
Model with survival as dependent													
* ICP* ≥ 20	0.17	(0.04–0.79)	0.024 *	0.07	(0.01–0.60)		0.015 *	0.10	(0.01–1.87)		0.123	0.871	
* CPP* ≤ 60	0.11	(0.01–1.08)	0.059	0.06	(0.00–3.65)		0.180	0.06	(0.00–2.35)		0.134	0.989	
* CPP* > 100	0.04	(0.01–0.27)	0.001 **	0.04	(0.00–0.81)		0.036	0.20	(0.01–3.00)		0.242	0.453	
* MAP* ≤ 80	0.80	(0.25–2.57)	0.701	0.74	(0.11–5.01)		0.759	0.24	(0.07–1.96)		0.244	0.594	
* MAP* > 120	0.12	(0.01–1.67)	0.115	0.19	(0.00–77.37)		0.589	0.78	(0.03–18.41)		0.877	0.685	
* SBP* ≤ 100	00.01	(0.00–0.10)	0.000 ***	0.00	(0.00–0.063)		0.001 **	0.00	(0.00–1.93)		0.079	0.822	
* SBP* > 180	0.03	(0.01–0.23	0.001 **	0.01	(0.00–0.51)		0.022 *	0.93	0.09 (0.09–9.97)		0.953	0.052	

Favorable outcome (GOSE 5–8) and survival (GOSE 2–8) at follow-up. OR odds ratio per one unit increase in %GMT. *p < 0.05, **p < 0.01, and ***p < 0.001

*OR* > 1 indicates that an increasing value of %GMT is associated with a higher odds of favorable outcome/survival. *OR* < 1 indicates that an increasing value of %*GMT* is associated with a lower odds of favorable outcome/survival

^d^Analyzed with multiple logistic regression for the physiological variable, age ≥ 65, and interaction (physiological parameter × age ≥ 65, *p* value for the interaction is presented

## Discussion

### Patient and management characteristics by age group

Patients ≥ 65 years of age constituted as much as 26% of all patients. Many of the patient characteristics found in relation to age were as expected. The most common cause of trauma in the elderly was fall accidents, which is in accordance with many other studies [7, 11, 13, 15, 16, 18, 20, 29, 35, 36]. There was a higher percentage of women among the elderly (29% vs 20%), which also was shown by Dams-O’Conner and coll., reporting an increasing proportion of women with increasing age (38.5% in 65–74 years, 50.4% in 75–84 years, and 62.2 in 85 years and older) [7]. The elderly more often had a medical history with previous diseases or injuries, e.g., 22.5% had a previous history of brain injury/disease, 58.3% hypertension/CVD, and 43% medicated with anticoagulants/antiplatelets. Similar results were found by Hawley and coll. showing that older TBI patients ≥ 65 had a recorded medical history in 80% and only 1.1% had no pre-existing medical condition [11]. The dominating injury type in the elderly was ASDH and diffuse injury was also less common according to the Marshall score. These findings are in line with that the dominating type of injury was falls in the elderly and that the elderly more often underwent craniotomy.

### Secondary insults/physiological variables—occurrence and association to age

The pattern of secondary insults/physiological variables differed by age. The elderly (≥ 65 years) spent a higher proportion of GMT with high *CPP*, high *MAP*, and high *SBP* and less degree of high *ICP*, low *CPP*, and low *MAP* (Table 5). Similar findings were also observed by Czosnyka and coll. [6].

In order to find out whether the observed difference between the age groups was explained by age independently, a multiple linear regression analysis was performed including age ≥ 65 years as a explanatory factor for the different predefined secondary insults/physiological variables. The analysis showed that age ≥ 65 years was an independent explanatory factor for higher %GMT with *CPP* > 100, *MAP* > 120, and *SBP* > 180 (Table 6). This finding may to some extent be explained by higher degree of hypertension and cardiovascular diseases in the elderly (Table 1). The crucial question is whether higher pressures may influence outcome in a negative way in the elderly.

### Secondary insults/physiological variables-relation to clinical outcome and interaction by age

The logistic regression analysis of outcome (favorable and survival) for all patients indicated that high %*GMT* with *ICP* > 20, *SBP* ≤ 100, *SBP* > 180, *CPP* > 100 not are beneficial. These findings, which may be summarized roughly as high *ICP*, low and high BP, and high *CPP* are bad, were not unexpected. Interestingly, when looking at the interaction analyses, the elderly had a higher AOR for favorable outcome.

Hence, blood pressure should probably be treated differently in younger and older patients. The finding that high blood pressures may be advantageous in elderly is supported by Utomo and coll. who found higher odds of independent living at 6 months for patients ≥ 65 years with a *SBP* on arrival at hospital in the range of 131–150 mmHg, compared to patients with *SBP* of < 130 mmHg[37].

*ICP* did not prove to be a significant predictor of outcome in the elderly. This finding should not be interpreted as if ICP is unimportant for outcome and does not need to be monitored in the elderly. Instead, this is probably an effect of the low burden of ICP insults thanks to effective detection and treatment. We have examined our material for events with %*GMT ICP* ≥ 25 and there was very few events in the elderly (median %*GMT* was 0.53, unpublished data). Monitoring of ICP in elderly with TBI is of importance and this has also been shown by You and coll. in a randomized trial of elderly with severe TBI who found lower in-hospital mortality and improved 6-month outcomes for the patients randomized to ICP monitoring [40]. We belive that extensive NIC monitoring is even more important in the elderly due to their increased vulnerability and this philosophy was clearly reflected in the observed numbers of elderly monitored in this study (Table 4), despite a larger proportion elderly using anticoagulants/antiplatelets.

### Limitations

This is a single-center study and the results may therefore be influenced by the local management applied. Thus, the results may not be completely generalizable. There was a selection bias since only patients judged to have a reasonable chance for favorable outcome were accepted for NIC. Treatment bias also needs to be considered since all patients were treated to avoid secondary insults and the % GMT at insult level was in low general.

Furthermore, complete multiple logistic regression analyses for assessing the influence of secondary insults on outcome could not be done (to adjust, e.g., sex, GCS at admission, and injury type) due to the relative small number of patients. It was however possible to study the age interaction.

### Conclusions

Elderly ≥ 65 years have different patterns of secondary insults/physiological variables, which to some extent is independently associated to age. When patients in all ages were analyzed, low %GMT with *CPP* > 100 and *SBP* > 180 were significant predictors of favorable outcome and high %GMT with *ICP* ≥ 20, *CPP* > 100, *SBP* ≤ 100, and *SBP* > 180 were positive predictors of death. The analysis of age interaction showed that patients ≥ 65 years differed and had a higher odds for favorable outcome and without a significant decrease in survival with large proportion of good monitoring time with *SBP* > 180.

This finding may indicate that blood pressure should be treated differently in younger and older patients. More TBI studies in the elderly are warrented to define specific guidelines regarding secondary insult definitions and optimal levels to target. Studies of pressure autoregulation and CPPopt are also desirable.

## Supplementary Information

Below is the link to the electronic supplementary material.

Supplementary file1 (DOCX 32 KB)

Supplementary file2 (DOCX 32 KB)

## Abbreviations

ASDH: Acute subdural hematoma
CPP: Cerebral perfusion pressure
CPPopt: Optimal cerebral perfusion pressure
CSF: Cerebrospinal fluid
CVD: Cardiovascular disease
CVP: Central venous pressure
DAI: Diffuse axonal injury
EDH: Epidural hematoma
EVD: External ventricular drainage
GCS M: Glasgow coma scale motor score
GMT: Good monitoring time
%GMT: Proportion of good monitoring time
GOSE: Glasgow outcome scale
ICP: Intra cranial pressure
MAP: Mean arterial pressure
NIC: Neurointensive care
OR: Odds ratio
SBP: Systolic blood pressure
TBI: Traumatic brain injury
