# Supplementary material for: Neurointensive care of traumatic brain injury in the elderly—age-specific secondary insult levels and optimal physiological levels to target need to be defined
Source: Acta Neurochir (Wien). 2021 Nov 10;164(1):117–28. doi: 10.1007/s00701-021-05047-z (PMC8761120; doi:10.1007/s00701-021-05047-z)
Supplement: Supplementary file 1 — Supplementary file1 (DOCX 32 KB) [file 701_2021_5047_MOESM1_ESM.docx]

# Neurointensive care of traumatic brain injury in the elderly – Age-specific secondary insult levels and optimal physiological levels to target needs to be defined.

Acta Neurochirurgica

# Samuel Lenell, MD; Anders Lewén, MD, PhD; Timothy Howells PhD; Per Enblad, MD, PhD.

Department of Neuroscience/Neurosurgery, Section of Neurosurgery, Uppsala University, Uppsala, Sweden.

**Corresponding author**

Samuel Lenell, M.D.

Department of Neuroscience, Section of Neurosurgery, Uppsala University

Uppsala University Hospital

SE-751 85 Uppsala

samuel.lenell@neuro.uu.se

**Supplementary information 1.** Age distribution
